# Design and optimization of a 16S microbial qPCR multiplex for the presumptive identification of feces, saliva, vaginal and menstrual secretions

**DOI:** 10.1111/1556-4029.15029

**Published:** 2022-03-30

**Authors:** Carolyn Lewis, Sarah J. Seashols‐Williams

**Affiliations:** ^1^ Integrative Life Sciences Doctoral Program Virginia Commonwealth University Richmond Virginia USA; ^2^ Department of Forensic Science Virginia Commonwealth University Richmond Virginia USA

**Keywords:** 16S ribosomal RNA gene, *Bacteroides uniformis*, classification regression tree model, forensic serology, *Lactobacillus crispatus*, presumptive body fluid identification, qPCR multiplex validation, quantitative polymerase chain reaction and qPCR, *Streptococcus salivarius*

## Abstract

Molecular methods for body fluid identification have been extensively researched in the forensic community over the last decade, mostly focusing on RNA‐based methods. Microbial DNA analysis has long been used for forensic applications, such as postmortem interval estimations, but only recently has it been applied to body fluid identification. High‐throughput sequencing of the 16S ribosomal RNA gene by previous research groups revealed that microbial signatures and abundances vary across human body fluids at the genus and/or species taxonomic level. Since quantitative PCR is still the current technique used in forensic DNA analysis, the purpose of this study was to design a qPCR multiplex targeting the 16S gene of *Bacteroides uniformis*, *Streptococcus salivarius*, and *Lactobacillus crispatus* that can distinguish between feces, saliva, and vaginal/menstrual secretions, respectively. Primers and probes were designed at the species level because these bacteria are highly abundant within their respective fluid. The validated 16S triplex was evaluated in DNA extracts from thirty donors of each body fluid. A classification regression tree model resulted in 96.5% classification accuracy of the population data, which demonstrates the ability of this 16S triplex to presumptively identify these fluids with high confidence at the quantification step of the forensic workflow using minimal input volume of DNA extracted from evidentiary samples.

**Highlights**

- A microbial qPCR multiplex was developed to classify forensically relevant body fluids.
- Three microbial species are used to distinguish feces, saliva, and vaginal/menstrual secretions.
- A classification regression tree model resulted in a 96.5% classification accuracy.
- The method could presumptively detect body fluids during DNA quantification.

## 1 INTRODUCTION

Body fluid identification (BFID) is the first step of the forensic DNA analysis workflow that can play a crucial role in story corroborations of suspects, victims, and/or witnesses. It can be useful for investigative leads and/or crime scene reconstruction. Equally important, it allows a DNA analyst to determine the best location to swab or cut to obtain a DNA profile from an evidentiary sample [1, 2]. Most methods currently used in forensic serology rely on enzymatic‐based tests that result in a color change that is interpreted and recorded by an analyst. Although these serological methods have been utilized for decades, there are well‐documented flaws associated with each [3, 4, 5, 6]. Therefore, there has been extensive research among the forensic community that addresses molecular‐based BFID methods, such as messenger RNA or microRNA analysis, DNA methylation, and microbial DNA analysis [7, 8, 9, 10, 11].

There are numerous studies that focus on microbiome‐based BFID, but mostly rely on 16S rRNA gene sequencing, as this is a gold standard in microbiome analysis [12, 13]. The 16S ribosomal subunit is specific to prokaryotes and is highly conserved within the same genus and species but has several hypervariable regions, all of which allow for taxonomical microbial classification [12]. An important consideration for forensic applications is the high cost and complex workflow of high‐throughput sequencing (HTS) methods. As of now, HTS is not standard in forensic DNA analysis due to cost, sample preparation time, hands‐on training requirements, and complicated back‐end bioinformatic analyses. To better align with the current DNA analysis workflow, real‐time (q)PCR methods for bacterial BFID have been proposed; however, most of these amplify multiple bacterial species to classify a single body fluid [14, 15, 16]. This could be problematic when developing a qPCR multiplex that can identify multiple body fluids due to the number of optical filters in qPCR instruments limiting how many sequences can be multiplexed in a single well. This approach would require the use of multiple reactions or wells, which would increase reagent cost and sample consumption compared with a single‐well assay.

The most successful microbiome‐based BFID studies focus on body fluids with high bacterial content, such as vaginal fluid, feces, and saliva [13, 15, 16, 17, 18, 19, 20, 21, 22]. One reported limitation is the inability to differentiate menstrual blood from vaginal secretions using microbial signatures, which could be useful information in a sexual assault case [21]. A major disadvantage is that some forensically relevant body fluids, particularly blood and semen, are difficult to characterize using microbial signatures because low bacterial cell counts often result in poor DNA yields, a problem when considering the often‐compromised nature of forensic evidence [13]. Several reports address these concerns by incorporating other types of molecular markers, such as messenger RNA; however, multiplexing challenges still apply to these integrated assays [17, 18, 23].

The purpose of this research was to design a qPCR multiplex targeting the 16S gene of three microbial species that are highly abundant in the respective body fluids—*Bacteroides uniformis* for feces, *Streptococcus salivarius* for saliva, and *Lactobacillus crispatus* for female intimate samples (vaginal fluid/menstrual blood). The main objective was to provide proof of concept that a single‐well microbial qPCR assay can presumptively identify more than one forensically relevant body fluid; therefore, differentiating between female intimate samples and identifying blood and semen were not primary goals of this study.

## 2 MATERIALS AND METHODS

### 2.1 Primer and probe design

Primers and probes were designed using default parameters in Beacon Designer 8 (Premier Biosoft, Palo Alto, CA). The same primer sequences from the SYBR Green mode were used to design the probes in TaqMan mode, which allowed for primer specificity testing before ordering/testing the hydrolysis probe. Primers and probes were ordered from Integrated DNA Technologies (IDT, Coralville, IA). Dual‐labeled hydrolysis probes were labeled with internal quenchers, HPLC‐purified and normalized to 100 μM in TE buffer. Sequence information for qPCR primers, probes, and respective targets are listed in Table 1.

**TABLE 1:** Microbial target and sequence information utilized in the 16S qPCR triplex. HPLC‐purified hydrolysis probes include 5′ reporters, internal, and 3′ quenchers

Microbial Target [Accession No.]	Forward Primer 5′‐3′	Reverse Primer 5′‐3′	Hydrolysis Probe 5′‐3′	Amplicon Length (bp)
*Lactobacillus crispatus* [MN744551]	CAGCAGTAGGGAATCTTC	CTGGTTGATTACCGTCAA	/ATTO550N/ACCTCTATC/TAO/CTTCTTCACCAACAACA/IAbRQSp/	145
*Bacteroides uniformis* [AP019724.1]	TAGCGGTGAAATGCTTAG	CATCGTTTACTGTGTGGA	/6FAM/CACGAAGAA/ZEN/CTCCGATTGCGAAG/IABkFQ/	136
*Streptococcus salivarius* [CP015282.1]	ATGCGTAGATATATGGAGG	CTACCAGGGTATCTAATCC	/SUN/CGAGCCTCA/ZEN/GCGTCAGTTACA/IABkFQ/	108

### 2.2 Sample collection and DNA extraction

Venous blood, menstrual blood, semen, saliva, feces, and vaginal samples were collected from 30 volunteers according to VCU’s Institutional Review Board–approved protocol for human subjects’ research (HM20002931). Menstrual blood, feces, saliva, and vaginal secretions were collected onto polyester swabs, while semen was collected in sterile plastic containers and aliquoted onto polyester swabs (50 μl). Venous blood was deposited onto polyester swabs by pricking the donor’s sterilized finger with a finger lancet. Swabs were placed in swab boxes and dried in at room temperature for at least 24 h prior to DNA extraction. The donations were collected between 2017 and 2019 for a biological sample registry, so they had been stored at room temperature for a comparable amount of time as they might in a forensic evidence room. Other considerations when selecting samples from the registry were antibiotic usage, equal number of male/female donors (when applicable), various ethnicities and ages, different days of the menstrual cycle, and at least seven‐day postcoital for intimate fluids (semen, vaginal fluid, and menstrual blood).

Whole swabs were extracted using the QIAamp® DNA Investigator Kit (Qiagen, Valencia, CA) following the “Isolation of Total DNA from Body Fluid Stains” protocol from the manufacturer. Reagent blanks were included in each batch of extractions to assess potential contamination. During cell lysis, no carrier RNA was added, and 20 μl of 1 M DTT was added to semen samples. After the 10‐min incubation at 70°C, residual liquid was collected by placing the swab in a DNA IQ™ spin basket (Promega, Madison, WI), returning it to the tube containing the lysate, and centrifuging at 3350 *g* for 5 min. Once the spin basket and swab were discarded, DNA purification was performed according to protocol without modification. All samples and reagent blanks were eluted in 50 μl of ATE buffer (Qiagen), and total DNA was quantified using the NanoDrop™2000 (Thermo Fisher Scientific) before storage at −80°C (data not shown).

### 2.3 qPCR

The designed multiplex is technically a qualitative‐PCR assay since the goal is not to quantify any microbial DNA in the sample, which would require analyzing known quantity standards alongside the questioned samples. However, synthetic DNA standards were used to validate the multiplex and could be used as positive controls or for quantification, if desired; therefore, the MIQE guidelines for qPCR reports were followed throughout the project [24].

Initially, each microbial target was evaluated as a single‐plex using gBlocks® Gene Fragments (IDT) as the qPCR standards. The gBlocks® were chosen as standards because there were no commercially available mock microbial community standards that included all three bacterial species of interest and because gBlocks® are manufactured (i.e., undergo extra quality control measures) for the purpose of validating qPCR assays. Sequences for each gBlock® standard are listed in Table S1. Each gBlock® was resuspended in TE buffer at 10 ng/μl and incubated at 50°C for 20 min, per manufacturer’s instructions. Ten‐fold serial dilutions were prepared at the concentration range of 5 pg/μl–0.05 fg/μl, aliquoted, and stored at −20°C. To validate the multiplex, the three gBlocks® sequences were pooled together at a concentration of 1 ng/μl before making ten‐fold serial dilutions to the optimized concentration range (5 pg/μl–0.05 fg/μl).

### 2.4 SYBR green assay

To ensure amplification of a single product, each primer set was evaluated first via a SYBR Green assay with melt curve analysis to ensure a single amplified product. Standards and no‐template controls were analyzed in triplicate using 6.25 μl of PerfeCTa SYBR Green SuperMix (2X) (VWR, Radnor, PA), 3.75 μl of nuclease‐free water, 0.25 μl (10 μM) of forward and reverse primers (Table 1), and 2 μl of standard for a 12.5 μl total reaction volume. Thermal cycling was conducted on the Applied Biosystems™ QuantStudio 6 Flex Real‐Time PCR System (Thermo Fisher Scientific, Waltham, MA) with PCR parameters set to 95°C for 3 min followed by 35 cycles of 95°C for 15 s, 58°C for 45 s, and 72°C for 30 s. With instrument default melt curve parameters. Raw data were analyzed in QuantStudio Software v1.3 (Thermo Fisher Scientific) with the baseline set at 1–6 cycles and threshold set to 0.02 for all targets.

### 2.5 Hydrolysis probe‐based assay

Once a single amplification product was confirmed in the SYBR Green assay, each target was individually evaluated in a probe‐based assay before multiplexing. After multiplex validation using gBlock® standards, the assay was tested using body fluid samples.

Each single‐plex qPCR reaction consisted of 5 μl of PrimeTime® Gene Expression Master Mix (2X) (IDT), 2.5 μl of nuclease‐free water, 0.5 μl of 20X assay mix, and 2 μl of standard for a total reaction volume of 10 μl. The 20X assay mixes for all targets were optimized to final determined concentrations of 400 nM for each primer and 200 nM for the probe. For the triplex reactions, 5 μl of PrimeTime® Gene Expression Master Mix (2X), 1.5 μl of nuclease‐free water, 0.5 μl of *L. crispatus* 20X assay mix, 0.5 μl of *B. uniformis* 20X assay mix, 0.5 μl of *S. salivarius* 20X assay mix, and 2 μl of sample or standard were combined for a 10 μl of total reaction volume. Thermal cycling parameters on the QuantStudio 6 Flex Real‐Time PCR instrument were set to 95°C for 3 min followed by 40 cycles of 95°C for 5 s and 60°C for 30 s in fast cycling mode. All six standards were analyzed in duplicate with no‐template controls on each plate. To conserve DNA extract, single technical replicates were run when testing body fluids; however, various extracts were tested multiple times throughout the validation to ensure repeatable results from body fluid samples. In QuantStudio Software v1.3, autobaseline and cycle threshold of 0.06 were applied initially during assay optimization, but the threshold was lowered to 0.04 for all targets during final validation and when evaluating body fluid specificity.

### 2.6 Data analysis

During assay optimization, standard curve metrics such as slope, Y‐intercept, and PCR efficiency (>90%) were analyzed for all targets, and technical validation was complete when the single‐plex and multiplex quantification cycle (Cq) values for each standard were within one cycle of one another. Raw data were exported from QuantStudio Software v1.3 and input into Microsoft Excel to calculate averages between technical replicates, replace a value of 40 for any “undetermined” result, and omit eight outliers that were greater than three standard deviations from the mean (yellow highlight in Table S2). Subsequent data analysis was performed in R version 4.1.1 (R foundation, Vienna, Austria). The data were randomly split into training and validation sets (70% and 30%, respectively) using the sample() function, and a classification regression tree (CART) model was created using the rpart package for each iterative analysis (15 analyses were conducted). Confusion matrices and classification accuracy percentages are reported for only one iteration of the validation dataset analysis. All data are reported as averages with standard deviations, where applicable.

### 2.7 Linear range of classification

The goal of the linear range of classification study was to determine the lowest DNA concentration at which the qPCR assay will accurately classify as the correct body fluid. Total DNA was quantified using the NanoDrop™2000 (Thermo Fisher Scientific) and averaged among the 30 donors of each body fluid in Microsoft Excel. Five donors of each body fluid were selected based on having DNA concentrations close to the calculated averages (blood = 4.13 ng/μl, menstrual blood = 65.19 ng/μl, semen = 22.04 ng/μl, feces = 117.16 ng/μl, vaginal fluid = 57.34 ng/μl, saliva = 19.04 ng/μl). Each DNA extract was serially diluted ten‐fold until the dilution concentration was less than the lower limit of the linear dynamic range (0.05 fg/μl), and then, qPCR was performed using the validated 16S triplex methods mentioned above. Data were analyzed using the trained CART model; however, since the confusion matrix output does not show which samples are misclassifying, the analysis was performed manually in Microsoft Excel for each sample.

## 3 RESULTS AND DISCUSSION

### 3.1 Assay validation

In both SYBR Green and probe‐based assays, there was no amplification detected in any of the negative controls, including extraction reagent blanks. Single peaks were observed in the SYBR Green melt curve analysis, which verified that there was only one PCR product for each primer pair (data not shown). The same primer sequences were then used for the probe‐based assay, in which various primer/probe concentrations and 5′ reporter dyes were evaluated during optimization. The final 16S triplex primer and probe concentrations for all three microbial targets were 400 nM (forward and reverse) and 200 nM, respectively. All reported data are representative of the 16S triplex at these concentrations. The 5′ reporter dyes were chosen based on having similar excitation and emission wavelengths as dyes that are in use in commercial STR multiplex kits, for example, the ATTO550 and SUN dyes (IDT) emit in the same filters as ABY and VIC (Thermo Fisher Scientific), respectively. These choices were designed to ensure that qPCR instruments would already be calibrated for the requisite probe emission spectra, which would ease implementation in forensic laboratories.

The slopes, amplification efficiencies, and *R* ^2^ values of the standard curves (Table 2) were all within the recommended ranges for qPCR assays [25]. The reported standard curve data in Table 2 are averaged across six experiments, demonstrating repeatability and reproducibility of the assay. The linear dynamic range was determined to be 5 pg/μl–0.05 fg/μl (Table S3 in Appendix S1). We acknowledge that the lower limit of this range is not the lowest limit of detection since each microbial target amplifies at approximately 28 cycles; however, lower concentrations were not tested to minimize reagent consumption. Additionally, we felt that six standards were sufficient to validate assay performance because the overall purpose is to obtain a raw Cq value rather than to quantify any microbial DNA from a sample. Although it is possible to quantify DNA of each microbial species using this assay, further research expanding the lower limit of detection would be required to quantify microbial DNA for forensic applications.

**TABLE 2:** Standard curve metrics across six experiments during the 16S triplex assay validation

Microbial Target	Slope	*Y*‐intercept	*R* ^2^	% Efficiency
*Lactobacillus crispatus*	−3.525 ± 0.05	2.719 ± 0.58	0.997 ± 0.002	92.2 ± 1.78
*Bacteroides uniformis*	−3.491 ± 0.03	2.433 ± 0.48	0.998 ± 0.002	93.4 ± 1.00
*Streptococcus salivarius*	−3.524 ± 0.03	2.549 ± 0.44	0.998 ± 0.002	92.2 ± 0.85

An important consideration during assay validation was equivalent amplification of the microbial DNA from forensic body fluid samples in a single‐plex assay when compared to the multiplex. To address this concern, multiple DNA extracts were tested for each target species both in single‐plex and multiplex reactions and verified that Cq values were within one cycle of each other (data not shown).

### 3.2 Evaluation of body fluid specificity

When evaluating raw data (Table S2), *S. salivarius* was detected in all body fluid samples at higher/similar abundances (lower Cq values) in saliva and menstrual blood compared with vaginal fluid and feces. As expected, *B. uniformis* and *L. crispatus* were most abundant in feces and vaginal/menstrual secretions, respectively. Of the three microbial species, detection of *L. crispatus* was the most variable, especially in vaginal fluid. One possible explanation for such high variability in vaginal fluid is PCR inhibition due to over‐input of template DNA (*L. crispatus* was undetected in nine vaginal samples). This is further discussed in the linear range of classification section. Another possible explanation is that only one *Lactobacillus* species was included in the assay compared with other microbial qPCR studies that target more than one species for accurate vaginal fluid identification [14, 16, 22]. Incorporating more markers into the assay would account for the diversity of the vaginal microbiome across females, but it also would increase reagent cost and consumption of DNA extract, which is something we wanted to avoid when designing the assay. An alternative solution could be to utilize the same approach as Doi et al. and design primers flanking a conserved region of 16S at the genus level of four *Lactobacillus* species [14].

### 3.3 Classification regression tree analysis

**FIGURE 1:**
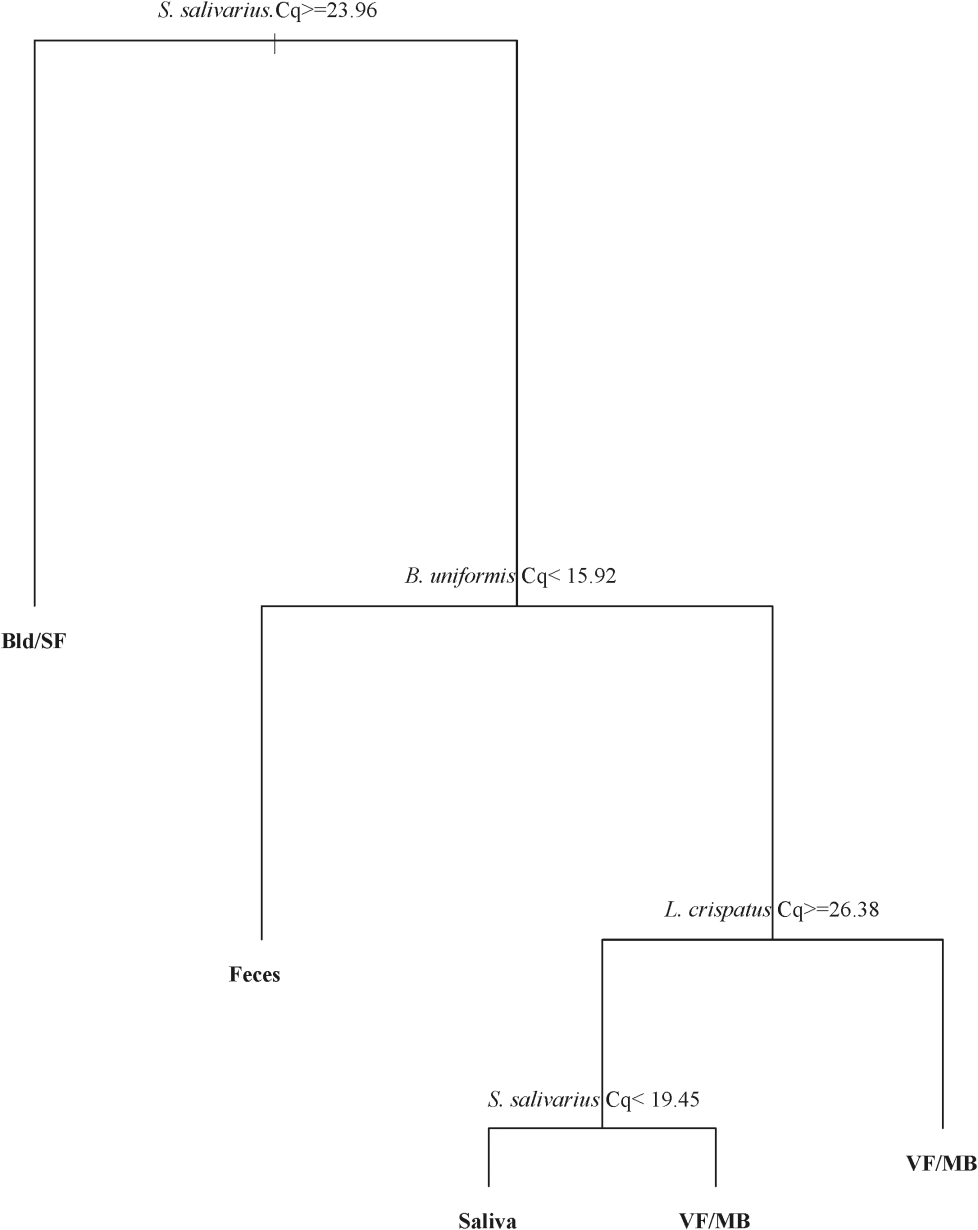
Classification Regression Tree (CART) model for classifying forensically relevant body fluids using a microbial 16S qPCR triplex when vaginal and menstrual secretions are grouped as female intimate samples (*n* = 30 donors of each body fluid, Bld/SF, blood/seminal fluid; VF/MB, vaginal fluid/menstrual blood)

It should first be noted that although blood and semen samples were evaluated in this study, the goal was not to differentiate between them using this assay, as they have low bacterial DNA yields [13] and were expected to have nearly undetectable values for all microbial species. For this reason, blood and semen were grouped together as “Bld/SF” in the dataset and were used to eliminate either fluid as the biological source in an unknown sample. Two regression trees were created—one with vaginal fluid (VF) and menstrual blood (MB) as separate fluids (Figure S1) and one as a combined group (VF/MB) (Figure 1). While it was not the original intent of this study to differentiate between vaginal/menstrual secretions, both were evaluated out of curiosity to see how each model would perform. The individual model resulted in an overall classification accuracy of 84% with 54.5% of MB samples misclassified. Four of the MB samples were classified as VF while two were classified as saliva (Table S4). Differentiation between VF and MB using the individual CART model was dependent on *S. salivarius* Cq value cutoff of 18.86 (Figure S1); however, a similar *S. salivarius* cutoff (Cq = 19.2) was used to distinguish between saliva and VF, which likely explains the observed misclassifications between VF, MB, and saliva (Table S4).

When female intimate sample data were combined, the tree plot (Figure 1) looked similar to that in Figure S1; however, the overall classification accuracy increased to 96.5% with only two samples (one saliva and one VF/MB) misclassified (Table 3). This demonstrated that combining female intimate samples in a dataset can increase classification accuracy. Furthermore, it supports the reported claim that it is difficult to differentiate vaginal fluid from menstrual blood using microbial signatures since both fluids originate from the same body cavity and can contain similar bacterial compositions at any point during the menstrual cycle [17, 18, 26]. This of course is somewhat appropriate given casework scenarios; however, as the project is still developmental in nature, additional future work will be required when classifying mixtures and distinguishing venous blood from menstrual secretions within a sample of mixed origin.

**TABLE 3:** Confusion matrix classifying body fluids using the 16S triplex in a trained Classification Regression Tree (CART) model

		Bld/SF	Feces	Saliva	VF/MB
	Bld/SF	**23**	0	0	0

Feces	0	**8**	0	0
Actual
Saliva	0	0	**6**	1

VF/MB	0	0	1	**17**

*Note:* A 96.5% overall classification rate was achieved when grouping female intimate samples together (VF/MB). Bold numbers indicate correct classifications (Bld/SF, blood/seminal fluid; VF/MB, vaginal fluid/menstrual blood) Predicted.

Importantly, 100% of fecal samples was correctly classified regardless of VF/MB grouping, and there were no misclassifications involving blood/semen samples. Saliva misclassifications were observed in both CART models, which could be due to higher‐than‐expected *S. salivarius* detection in other body fluids thus negatively impacting its anticipated saliva specificity. Another possible explanation is that *S. salivarius* primers and probe were designed at the species level, and differentiation among *Streptococcus* species in saliva has been reportedly more difficult using 16S compared with other genes [19, 27]. If this is true, there could be other *Streptococcus* species amplifying, which would result in lower Cq values. This could possibly explain why *S. salivarius* was detected in all donors in every body fluid sample, whereas *L. crispatus* and *B. uniformis* were undetected in at least one donor of all body fluids (Table S2). These results are contrary to what has been reported in the literature, which state that *S. salivarius* was not detected in other body fluids; however, one group did not examine feces [28], and different methodologies were used, such as loop‐mediated isothermal amplification (LAMP), reverse transcription LAMP, or direct PCR combined with immunochromatographic strip [28, 29, 30]. Importantly, none of these studies amplified 16S, which supports the previous statement that the 16S rRNA gene may not be the best target for saliva identification, especially at the species taxonomic level. Since saliva is commonly present on crime scene evidence, incorporating a more specific saliva marker into the proposed qPCR multiplex may be useful for forensic casework implementation.

### 3.4 Linear range of classification

The goal of this study was to determine at which ten‐fold dilution of DNA extract the body fluid will classify correctly using the grouped female intimate CART model. All saliva samples could only be accurately classified when the DNA extract was input into the qPCR reaction (Table S5). The lowest DNA concentrations quantified via UV‐spectrophotometry were observed in saliva compared with vaginal/menstrual secretions and feces (data not shown); therefore, it was expected that ten‐fold dilutions of saliva extracts would not yield correct classification results. It should be noted that, unless otherwise stated, any dilution that was correctly classified was also correctly classified in the DNA extract; for example, vaginal fluid and feces were correctly classified in DNA extracts of all five donors but only in the first dilution (D1) for three donors (Table S5).

There were no fluids that classified correctly beyond the second dilution (D2), except for MB, which was still accurately classified in the second dilution for four out of five donors (Table S5). There was 1‐MB sample that could only be classified in the DNA extract; however, *L. crispatus* was never detected in that individual throughout the project. This could be due to a lower abundance (or absence) of that *Lactobacillus* species in the vaginal microbiome of that particular donor, which could be a result from a clinical infection, such as bacterial vaginosis. Alternatively, that individual may naturally have decreased *L. crispatus* abundance during their menstrual cycle. These are reasons why large population sizes of various demographics should be evaluated for any microbial assay, so that intraperson and interperson variability can be assessed and so that false positive/negative rates can be described.

An interesting observation in this study was that in two vaginal samples, *L. crispatus* was undetected in the DNA extract yet was detected in subsequent dilutions, which supports the previous hypothesis that inputting too much DNA template into the reaction may lead to PCR inhibition. This only affected classification accuracy in one of the VF samples, where classification was only achieved in the first dilution but not in the DNA extract. Of note, the proposed CART analysis method evaluates raw Cq values, so it is expected that dilution of a DNA extract will equally dilute all DNA in the sample; therefore, a different analysis method or data normalization may be useful for compromised evidence samples.

## 4 CONCLUSIONS

In this study, a microbial 16S qPCR multiplex was proposed, which can correctly classify vaginal fluid, menstrual blood, feces, and saliva with 84% overall accuracy through classification regression tree analysis. When female intimate samples were grouped together in the CART model, overall accuracy increased to 96.5%. All observed misclassifications involved saliva samples, which indicates that different saliva‐specific markers or redesign of the *Streptococcus* primers may increase the assay’s ability to correctly classify saliva. Importantly, high accuracy was achieved using forensically relevant dried samples of appropriate volumes that had been stored at room temperature for an extended period of time, which suggest that bacterial signatures remain stable enough to characterize dried body fluids via qPCR. The assay was developed for use on a qPCR instrument with five optical filters, and only four are used in the multiplex (three for microbial targets and one for passive reference dye); therefore, it is possible to include one more primer set and remain a single‐well assay. An internal positive control should be considered whether synthetic standards are not run alongside unknown samples. Alternatively, the incorporation of a human specific primer set would allow for human DNA quantification and confirm that the sample is of human origin. In this case, the method would not require any additional steps in the current DNA workflow and would consume minimal volume (1–2 μl) of DNA extract, which would allow for easy implementation into forensic laboratories.

This study demonstrated proof of concept that presumptive identification of multiple body fluids can be achieved using a single‐well qPCR assay that does not rely on amplification of multiple microbial species per body fluid for accurate classification of feces, saliva, and vaginal/menstrual secretions. This is of importance because there are no current in‐use serology tests that identify vaginal/menstrual secretions or feces, both of which could provide useful information during sexual assault investigations. Future research should address the incorporation of nonmicrobial biomarkers for blood and semen, since there are two of the most common fluids found on crime scene evidence. Body fluid mixtures, compromised or degraded samples, differences in microbial signatures across and within populations, and other forensic considerations, such as antibiotic usage, species specificity, and substrate comparisons, should also be addressed in future work as full characterization for developmental validation purposes.

## Supporting information


Appendix S1

Table S2
