# Supplementary material for: Design and optimization of a 16S microbial qPCR multiplex for the presumptive identification of feces, saliva, vaginal and menstrual secretions
Source: J Forensic Sci. 2022 Mar 30;67(4):1660–7. doi: 10.1111/1556-4029.15029 (PMC9310585; doi:10.1111/1556-4029.15029)
Supplement: Supplementary file 1 — Appendix S1 [file JFO-67-1660-s002.docx]

| **Microbial Target & [Accession No.]** | **gBlock**^®^ **Gene Fragment Sequence**  5’ – 3’ |
| --- | --- |
| *Lactobacillus crispatus*  [MN744551] | CCCAAACTCCTACGGGAGGCAGCAGTAGGGAATCTTCCACAATGGACGCAAGTCTGATGGAGCAACGCCGCGTGAGTGAAGAAGGTTTTCGGATCGTAAAGCTCTGTTGTTGGTGAAGAAGGATAGAGGTAGTAACTGGCCTTTATTTGACGGTAATCAACCAGAAAGTCACGGCTAACTACGTGCCAGC |
| *Bacteroides uniformis*  [AP019724.1] | AGGCAGGCGGAATTCGTGGTGTAGCGGTGAAATGCTTAGATATCACGAAGAACTCCGATTGCGAAGGCAGCTTGCTGGACTGTAACTGACGCTGATGCTCGAAAGTGTGGGTATCAAACAGGATTAGATACCCTGGTAGTCCACACAGTAAACGATGAATACTCGCTGTTTGCGATATAC |
| *Streptococcus salivarius*  [CP015282.1] | GAATTCCATGTGTAGCGGTGAAATGCGTAGATATATGGAGGAACACCGGTGGCGAAAGCGGCTCTCTGGTCTGTAACTGACGCTGAGGCTCGAAAGCGTGGGGAGCGAACAGGATTAGATACCCTGGTAGTCCACGCCGTAAAC |

TABLE S1 IDT gBlock^®^ Gene Fragment sequences used as qPCR standards during the 16S triplex validation

TABLE S2 Cycle threshold (Cq) values for each body fluid sample tested with the 16S triplex (see Table S2 exported as separate Excel file)

TABLE S3 Example of standard curve metrics and raw data for 16S microbial triplex demonstrating equal amplification of all three targets in a single well (Conc.=DNA concentration of pooled standard of IDT gBlocks^®^ Gene Fragments)

1. *Lactobacillus crispatus*

| **Target** | **Reporter** | **Conc.**  **(pg/µL)** | **Cq Mean** | **SD** | **Slope** | **Y-int** | **R^2^** | **Efficiency %** |
| --- | --- | --- | --- | --- | --- | --- | --- | --- |
| L.crispatus_16S_ATTO550 | ABY | 5 | 10.181 | 0.47 | -3.534 | 2.062 | 0.999 | 91.86% |
|  |  | 0.5 | 13.947 | 0.12 |  |  |  |  |
|  |  | 0.05 | 17.166 | 0.02 |  |  |  |  |
|  |  | 0.005 | 20.559 | 0.06 |  |  |  |  |
|  |  | 0.0005 | 24.265 | 0.01 |  |  |  |  |
|  |  | 0.00005 | 28.049 | 0.08 |  |  |  |  |

1. *Bacteroides uniformis*

| **Target** | **Reporter** | **Conc.**  **(pg/µL)** | **Cq Mean** | **SD** | **Slope** | **Y-int** | **R^2^** | **Efficiency %** |
| --- | --- | --- | --- | --- | --- | --- | --- | --- |
| B.uniformis_16S_FAM | FAM | 5 | 9.876 | 0.08 | -3.483 | 1.821 | 0.999 | 93.70% |
|  |  | 0.5 | 13.413 | 0.07 |  |  |  |  |
|  |  | 0.05 | 16.760 | 0.13 |  |  |  |  |
|  |  | 0.005 | 20.072 | 0.01 |  |  |  |  |
|  |  | 0.0005 | 23.717 | 0.09 |  |  |  |  |
|  |  | 0.00005 | 27.410 | 0.11 |  |  |  |  |

1. *Streptococcus salivarius*

| **Target** | **Reporter** | **Conc.**  **(pg/µL)** | **Cq Mean** | **SD** | **Slope** | **Y-int** | **R^2^** | **Efficiency %** |
| --- | --- | --- | --- | --- | --- | --- | --- | --- |
| S.salivarius_16S_SUN | VIC | 5 | 10.079 | 0.14 | -3.518 | 1.975 | 0.999 | 92.43% |
|  |  | 0.5 | 13.736 | 0.13 |  |  |  |  |
|  |  | 0.05 | 17.090 | 0.11 |  |  |  |  |
|  |  | 0.005 | 20.375 | 0.03 |  |  |  |  |
|  |  | 0.0005 | 24.043 | 0.05 |  |  |  |  |
|  |  | 0.00005 | 27.861 | 0.01 |  |  |  |  |

TABLE S4 Confusion matrix classifying body fluids using the 16S triplex in a trained Classification Regression Tree (CART) model. An 84% overall classification rate was achieved when analyzing vaginal and menstrual secretions as individual body fluids. Bold numbers indicate correct classifications (Bld/SF=blood/seminal fluid, VF=vaginal fluid, MB=menstrual blood)

**Predicted**

|  |  | Bld/SF | Feces | MB | Saliva | VF |
| --- | --- | --- | --- | --- | --- | --- |
|  | Bld/SF | **19** | 0 | 0 | 0 | 0 |
| **Actual** | Feces | 0 | **9** | 0 | 0 | 0 |
|  | MB | 0 | 0 | **5** | 2 | 4 |
|  | Saliva | 0 | 0 | 0 | **6** | 1 |
|  | VF | 0 | 0 | 1 | 1 | **8** |

TABLE S5 Linear range of classification for the 16S triplex demonstrating at which DNA dilution the body fluid remained correctly classified. Five donors of each were serially diluted ten-fold based on total DNA concentration and evaluated using the CART model with female intimate samples grouped together (VF=vaginal fluid, MB=menstrual blood, D=dilution)

**Body Fluid (n=5 donors)**

|  |  | Saliva | Feces | VF | MB |
| --- | --- | --- | --- | --- | --- |
| **Dilution** | Extract | 5/5 | 5/5 | 4/5 | 5/5 |
|  | D1 | 0 | 3/5 | 3/5 | 4/5 |
|  | D2 | 0 | 0 | 0 | 4/5 |

FIGURE S1 Classification Regression Tree (CART) model for the 16S triplex data when vaginal fluid (VF) and menstrual blood (MB) are analyzed as separate biological fluids (Bld/SF=blood/semen)
